# Saudi Electronic Caries Assessment Tool (SECAT) Development: Mixed Methods Feasibility Study

**DOI:** 10.3390/healthcare13050483

**Published:** 2025-02-23

**Authors:** Haya Alayadi, Arwa Talakey, Tourkiah Alessa, Abdulaziz Aldhalaan

**Affiliations:** 1 Dental Health Department, College of Applied Medical Sciences, King Saud University, Riyadh 11451, Saudi Arabia; 2 Department of Periodontics and Community Dentistry, College of Dentistry, King Saud University, Riyadh 11451, Saudi Arabia; aatalakey@ksu.edu.sa; 3 Department of Biomedical Technology, College of Applied Medical Sciences, King Saud University, Riyadh 11451, Saudi Arabia; talessa@ksu.edu.sa; 4 Cloud Software Computing Development, General Department of Development, Cloud Computing, National Information Center, Saudi Data and AI Authority, Riyadh 12382, Saudi Arabia; abdulaziz1928@gmail.com

**Keywords:** dental caries, mobile applications, tele-dentistry, health surveys, Saudi Arabia

## Abstract

**Background:** Dental caries is a significant public health challenge globally, particularly acute in Saudi Arabia’s remote areas with limited healthcare access. Traditional paper-based methods for recording epidemiological data have limitations in data collection, storage, and sharing, highlighting the need for mobile solutions to enhance dental surveillance in resource-limited settings. **Objective:** To develop and evaluate the Saudi Electronic Caries Assessment Tool (SECAT), a mobile application designed for collecting dental caries data in remote locations, following a user-centered design approach. **Methods:** This mixed-methods feasibility study was conducted in three stages: (1) requirement gathering through semi-structured interviews with 12 dental professionals to explore experiences and needs; (2) development of the SECAT application using Flutter frontend and Supabase backend; and (3) evaluation through usability testing with 18 clinicians and heuristic study with five domain experts. **Results:** The usability study revealed an 82% overall satisfaction rate among clinicians, with 78% strongly endorsing the application’s user-friendly features. Expert evaluation highlighted the application’s utility for remote areas and offline functionality. Key improvements implemented based on feedback included an automated tooth recognition system, enhanced visualization protocol, and integration of clinical metrics. Primary limitations identified included the need for improved color contrast and individual-level calculations of dental indices. **Conclusions:** The SECAT application demonstrated high satisfaction and acceptability among dental health providers for collecting examination data in remote locations. The preliminary evaluation identified both limitations and positive aspects, particularly regarding utility in areas with limited internet connectivity. The SECAT mobile application could be valuable aid for caries assessment in remote places and also in school and community-based dental health programs.

## 1. Introduction

Dental caries is defined as a “biofilm-mediated, sugar-driven, multifactorial, dynamic disease that results in the phasic demineralization and remineralization of dental hard tissues” [1]. It remains a significant public health challenge globally, affecting 60–90% of the population worldwide [2,3]. Untreated dental caries can cause discomfort, infection, and swelling, leading to emergency visits to the dentist, particularly in young children [4]. In Saudi Arabia, a systematic review found that caries prevalence from 1999 to 2019 reached up to 100% in primary teeth and 99% in permanent teeth [5]. This issue is particularly acute in remote and underserved areas with limited access to oral healthcare services [6]. Accurate and timely data collection on caries prevalence and severity is crucial for informing public health interventions and resource allocation [7]. However, conducting oral health surveys in remote locations poses logistical challenges related to transportation of equipment, data recording, and transmission of information [8].

Traditional paper-based methods for recording epidemiological data have limitations in terms of data collection, storage, and sharing. With advancements in mobile technologies and their growing use in healthcare systems, there is a need for mobile applications to improve epidemiologic surveillance in dentistry [3]. A previous systematic review by Estai et al. [9] have highlighted the use of tele diagnosis in caries diagnosis and scoring, but those technology were implemented in limited resources set-up.

Mobile health (mHealth) applications have shown promise for enhancing intervention, data collection and clinical decision support in various healthcare fields [10,11]. These health applications must be designed with their target users’ needs in mind to be simple to use and deemed beneficial [12]. Nonetheless, the mHealth industry continues to underutilize human or user-centered design thinking methodologies [13]. Premature implementation of unapproved mHealth applications may restrict their use in a healthcare system currently plagued by substandard outcomes and high costs [12]. The quality of the mHealth applications developed is adversely affected when end-users and clinicians are not involved in the design and development stages. It would be beneficial to include users earlier in the development process and identify ways to combine end-user requirements with evidence-based content in order to create applications that are both high-quality and useful [14]. On the contrary, developing and implementation of mHealth applications is not without limitations. Adaptability, usability, interactivity, application administration (authorization, data protection), and evolving health requirements are identified as possible major obstacles to the adoption of a new technology [15].

The number of mHealth applications with a user-centered design intended for patients and the general public has increased significantly in recent years. However, not much research has been done on applications for healthcare providers as end users [16], specifically in dental field. In the current study, we developed and evaluated a mobile application called the Saudi Electronic Caries Assessment Tool (SECAT) for collecting dental caries data in remote locations in Saudi Arabia. The application development project utilized a user-centered design approach, considered as one of the most suitable approaches to health application development, ensuring to meet potential users’ preferences and needs [17]. The study followed a similar methodological approach as used by Alhodaib et al. (2020) in developing a mobile clinical decision support system for diabetes management [18]. The previous study demonstrated the value of incorporating end-user perspectives throughout the design process to enhance usability and acceptability.

The SECAT application was designed to standardize caries data collection, improve efficiency, and enable real-time data transmission from field sites to centralized databases. Key features included the following: (1) A user-friendly interface for inputting dental examination data based on World Health Organization (WHO) criteria [19], (2) local data storage on mobile devices for use in areas with limited internet connectivity, (3) synchronization capabilities to transfer data to a central server when internet is available, (4) built-in analysis tools for calculating caries indices and generating basic reports.

This feasibility study involved three key stages: (1) gathering requirements from potential end-users, (2) developing a prototype application, and (3) evaluating its impact, usability and acceptability among dental professionals [18].

The findings from this project will guide future iterations of the SECAT application and provide insights into the potential of mHealth solutions to enhance oral health surveillance and care delivery in remote locations. Additionally, lessons learned may inform the development of similar tools for use in other resource-limited settings globally. Thus, the aim of this study was to conduct a feasibility project developing and evaluating a SECAT mobile application for collecting data in remote locations.

## 2. Materials and Methods

### 2.1. Ethical Considerations

This study was carried out in accordance with the code of ethics of the World Medical Association (Declaration of Helsinki). Ethical approval was obtained from the institutional review board at King Saud University (IRB Number E-24-9094), which approved all stages of the study. Informed consent was obtained from all participants of this study.

### 2.2. Copyright © 2024

All rights of Saudi Electronic Caries Assessment Tool (SECAT) reserved through copyright certificate No. 24-12-16735072. This application, including all its features, content, and associated documentation, is protected by Saudi Arabian and international copyright laws. No part of this application may be reproduced, distributed, or transmitted in any form or by any means without the prior written permission of the copyright holders.

Developed at King Saud University, Riyadh, Saudi Arabia.

### 2.3. Research Framework

The current research was in compliance with the main stages of the user-centered design approach, which is a generic, multidisciplinary, and user-oriented approach to software development, where the potential users, their preferences, their requirements and needs at the center of attention [18].

The three main phases of the user-centered design framework—a general, multidisciplinary, and user-oriented approach to software development that places the intended users, their needs, and their requirements front and center—formed the basis of the current research study.

### 2.4. Stage 1: Requirements Gathering

#### 2.4.1. Study Design

The initial step of the current research included an exploratory qualitative method to explore participants’ current or previous experiences with career-related mobile application employed as an assessment tool and their attitudes toward using technology, with a focus on desired features and needs in career-related mobile application. This study was done using semi-structured interviews (face-to-face) with both dentist and dental hygienist.

#### 2.4.2. Setting and Participants

The qualitative study was conducted in King Khalid University hospitals (KKUH) and College of Applied Medical Sciences (CAMS) clinics, Riyadh, Saudi Arabia.

Convenience sampling was used to recruit participants via flyers or posters. Participants responded via phone or email and were sent an information sheet. Suitable times were then arranged for the interviews. A consent form was also obtained before commencing each interview. The eligibility criterion for participants was only dentists and dental hygienists with at least two years of work experience. Accordingly, the study initially aimed for a minimum of 10 dentists and dental hygienists, but employed a flexible approach to continue recruiting until data saturation was achieved [20,21]. The researchers determined that saturation had been reached after conducting 12 interviews, at which point they concluded the recruitment process.

#### 2.4.3. Data Analysis

Descriptive statistics were conducted for the relevant quantitative data. All qualitative interviews were recorded, transcribed, and then checked for accuracy against the audio files before being translated. Thematic analysis was utilized to analyze interviews data using NVivo software (v.14, Lumivero, Denver, CO, USA). To enhance the objectivity of data interpretation and reduce potential bias, the research team sought guidance from an expert in qualitative research methodologies regarding the study’s implementation and analytical processes.

### 2.5. Stage 2: Design and Development of the SECAT Application

#### 2.5.1. Requirements

The feedback from the dentists and dental hygienists at the previous step, in addition to other requirements collected from the previous literature, and suggestions from two dentists’ consultants were considered during step 2.

#### 2.5.2. Design and Development

**Figure 1:**
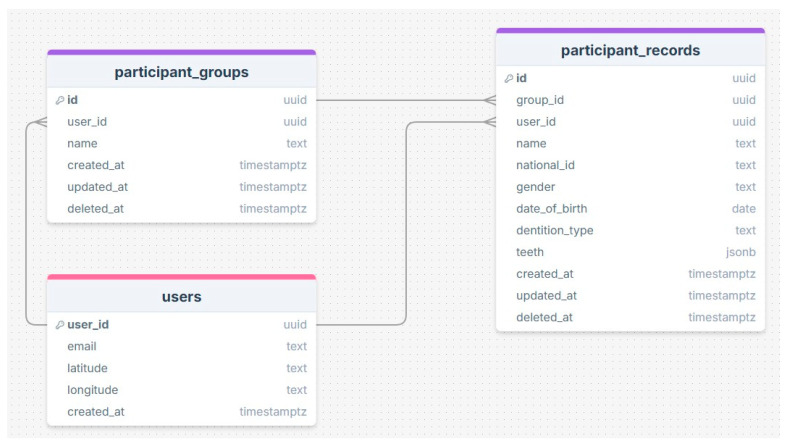
SECAT Database Design.

The SECAT app was developed by a collaborative team consisting of a computer science professional, consultant dentist, health informatics consultant, and dental health consultant. The development process included two key parts: design and coding (Appendix A-Flow chart, and Figure 1: Database design). The WHO coding system for dental assessment was followed in developing the SECAT application, ensuring adherence to standardized and accurate evaluations (WHO, 2013 [19]).

This SECAT application was designed using a flutter frontend and supabase for the backend, which is an open-source, offering application programming interface (API) capabilities and a postgreSQL database with real-time data synchronization and API capabilities. The supbase cloud-hosted version was used temporarily until finalizing the last stage of the application. To adhere with security best practices, the cloud-hosted version enables secure transport encryption, Hypertext Transfer Protocol Secure (HTTPS) and row-level security to only allow authenticated/authorized user access or modify data.

### 2.6. Stage 3: Evaluation Stage

#### 2.6.1. Research Design

This phase of the study employed a mixed-methods approach to evaluate the application usability and acceptance. The research design incorporated two methods: a usability test and a satisfaction questionnaire [22] (Appendix B). The objective was to assess the application’s impact, gauge its acceptability among users, and identify potential areas for improvement. This comprehensive evaluation strategy aimed to provide both quantitative and qualitative insights into the application’s performance and user experience, thereby informing future refinements and enhancements.

#### 2.6.2. Recruitment

The evaluation framework for SECAT encompassed multiple methodological approaches, incorporating both user-centric and expert-based assessment protocols. The evaluation strategy consisted of two primary components: a usability assessment for potential users, and a heuristic evaluation conducted for domain experts. The participant sample was strategically divided into two cohorts through stratified sampling. The first cohort comprised dental healthcare practitioners (n = 18), including dentists and dental hygienists, who provided insights from a clinical implementation perspective. The second cohort consisted of academic domain experts (n = 5), selected based on their theoretical expertise and research credentials in relevant disciplines. This bifurcated evaluation approach enabled the integration of both practical/clinical insights and theoretical/academic perspectives, facilitating comprehensive data triangulation.

The usability evaluation comprised 18 clinicians who participated in a multi-method assessment protocol. The protocol consisted of simulation-based scenarios, think-aloud protocols, and usability questionnaires. Individual interviews were conducted face-to-face, during which participants engaged with two simulation-based case scenarios (see Appendix C). These scenarios, developed by the principal investigator, required participants to interact with the SECAT application through data input tasks. Concurrent think-aloud protocols were employed, wherein participants verbalized their cognitive processes while completing the tasks, with the principal investigator documenting key observations. This evaluation phase was designed to assess the application’s utility in supporting healthcare providers’ workflow. Following the completion of the scenarios, participants completed a usability questionnaire to evaluate the application’s effectiveness and user experience.

The participant recruitment process leveraged an existing pool of dental professionals who had previously contributed to the interview phase and expressed interest in continued involvement. Electronic correspondence was dispatched to 12 dental practitioners, encompassing both dentists and dental hygienists. The final cohort comprised five participants who consented to engage in the heuristic evaluation protocol. The methodology entailed an initial demonstration of the application, followed by a hands-on exploration phase during which experts were tasked with examining the interface and technical components while verbalizing their assessments. This protocol enabled the contemporaneous documentation of user engagement patterns and cognitive workflows. To augment the observational data, researchers implemented semi-structured inquiries through open-ended questions, thereby eliciting nuanced perspectives regarding the application’s efficacy. This methodological triangulation facilitated a thorough assessment of both the user interface design and functional attributes of the software platform.

#### 2.6.3. Statistical Analysis of Usability Study

The quantitative analysis of usability study feedback employed a weighted scoring system. The evaluation utilized a summative assessment methodology, wherein participants’ responses were measured on a 5-point Likert scale, aggregated with weighted values, and subsequently the percentage was calculated to normalize a standardized 100-point scale. Descriptive statistics through measures of frequency (count and percentage) were used to describe the sample distribution and characteristics using Microsoft Excel (Microsoft, Redmond, WA, USA).

## 3. Results

### 3.1. First Step: Requirements Gathering

#### 3.1.1. Participant Characteristics

The interviews were conducted with a total of 12 dentists and dental hygienists from KKUH and CAMS clinics in Riyadh, Saudi Arabia regarding any current or previous experiences with career-related mobile application employed as a caries assessment tool, their attitudes toward using technology, with a focus on desired features and needs in career-related mobile application. The majority of the sample were female (n = 8) with average age of 35 years and average work experience of 7 years. Participant characteristics are summarized in Table 1.

#### 3.1.2. Interview Findings

**Figure 2:**
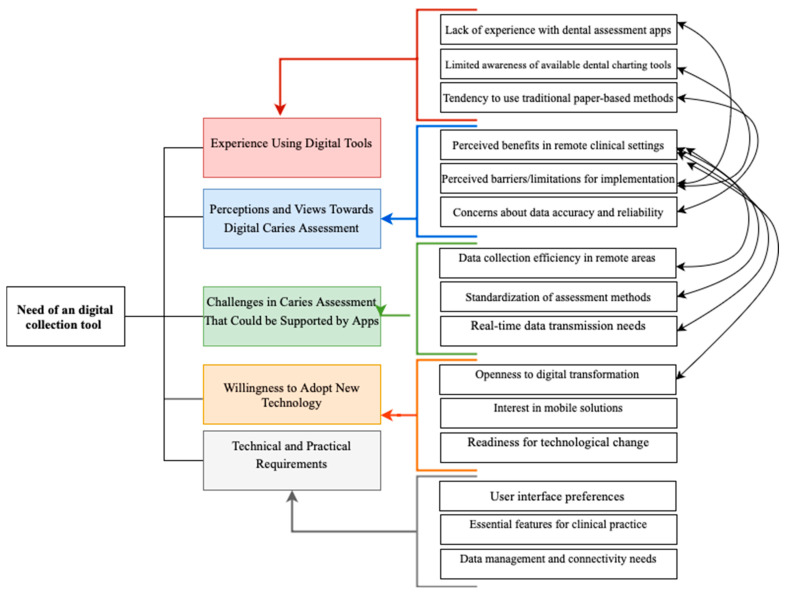
SECAT themes identified by the interview and their relationships.

The interview session with dentists and dental hygienists identified different themes categorized in seven main areas: current practices in caries assessment, technology adoption and comfort level, desired features for caries assessment application, perceived impact on workflow, user experience and usability factors, barriers and limitations to application adoption, willingness to adopt new technology. The themes identified by the interview and their relationships are shown in Figure 2. Examples of dentists and dental hygienists’ answers that helped formulate the conclusions are provided in Appendix D.

### 3.2. Second Step: Design and Development of the Mobile, Clinical Decision Support Application

**Figure 3:**
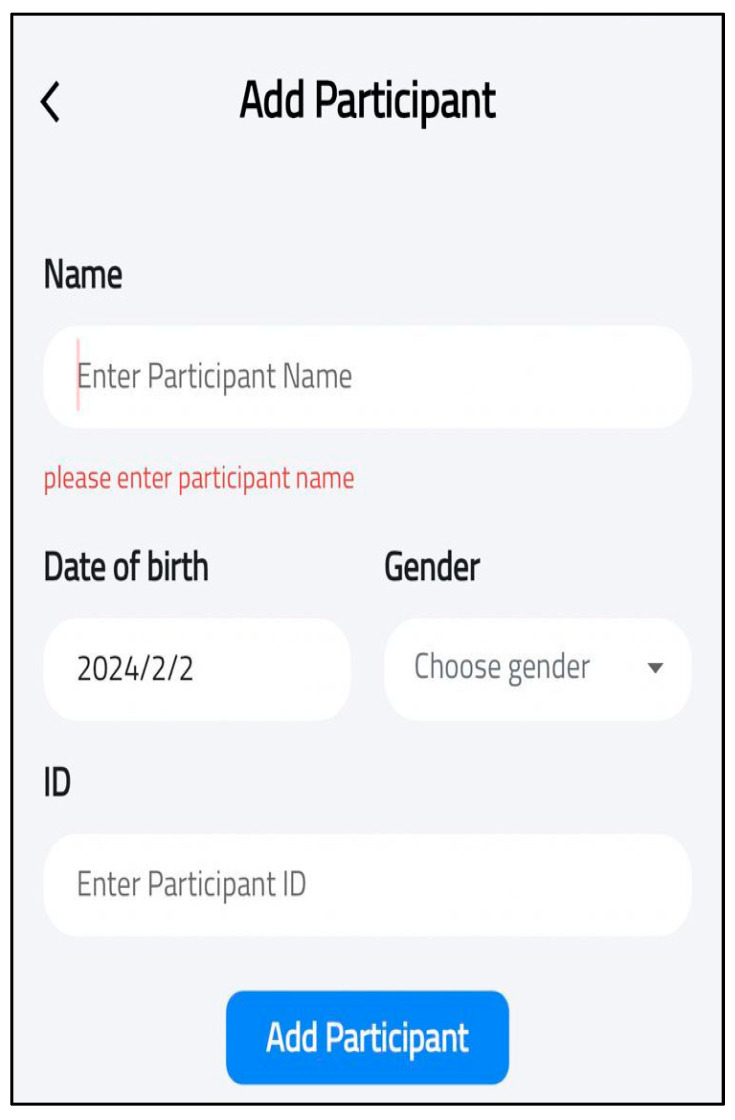
Add patient details.

**Figure 4:**
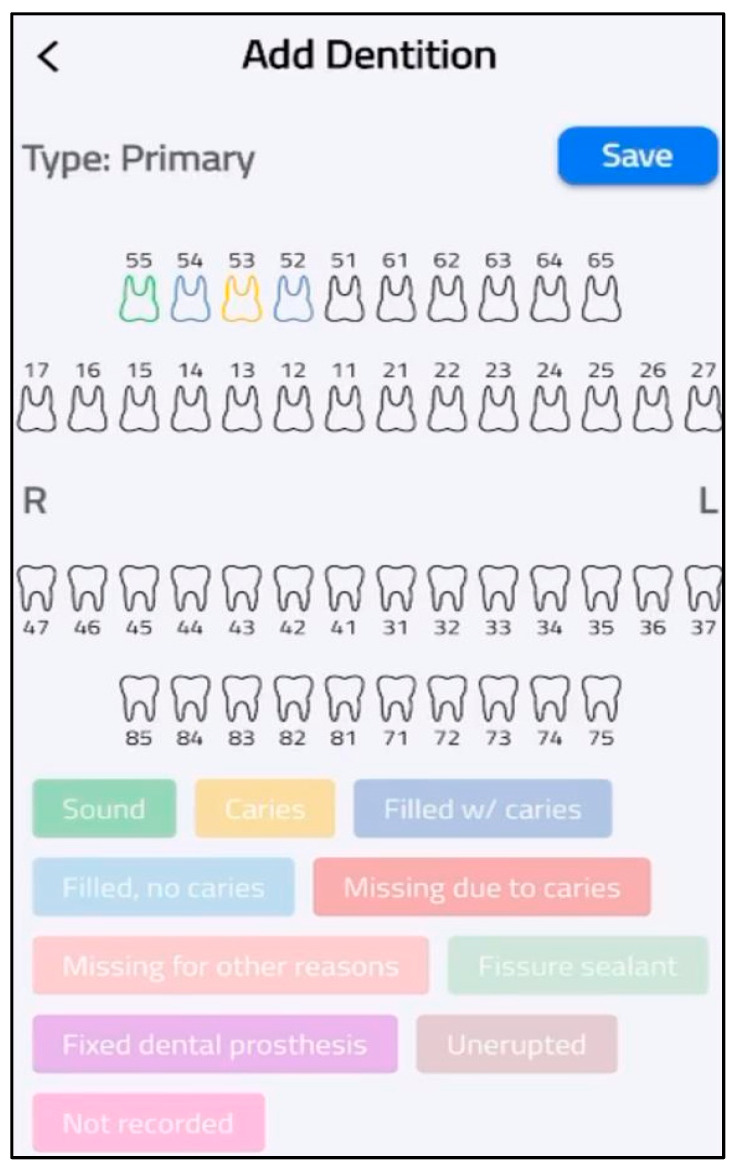
Dental examination.

At this phase, the SECAT smartphone app was developed. The aim of this application was to standardize caries data collection and enable real-time data transmission from the application to centralized databases. The application offers an easy-to-follow interface and has four main features: participant entry, dentation examination, clinical information and data storage. The first feature allows users to add and save each participant’s details (ID, gender., etc.) for each application user, as shown in Figure 3. The second feature allows users to select the appropriate dentition type of each participant (e.g., primary, permanent or mixed) as well as entering dental examination data according to WHO criteria, as shown in Figure 4. The application also includes main clinical information that provides information on calculating caries indices and summarizing basic findings (e.g., missing teeth). The final feature is local data storage on mobile devices which can allow data entry at areas with limited internet connectivity, and sync onto central server once connection is restored.

### 3.3. Third Step: Evaluation

#### 3.3.1. Participant Characteristics

This study comprised two distinct studies of participant recruitment: one that included clinicians and another that included an expert. In the usability study, 18 clinicians were enlisted, consisting of ten females and eight males; nine were dental hygienists and nine dentists, with a mean age of 34 years. In the acceptability study, the expert group involved a group of five participants, three dentists and two dental hygienists, with a gender distribution of two males and three females, and an average age of 42 years. Participant characteristics are summarized in Table 2.

#### 3.3.2. Usability Study Results

**Figure 5:**
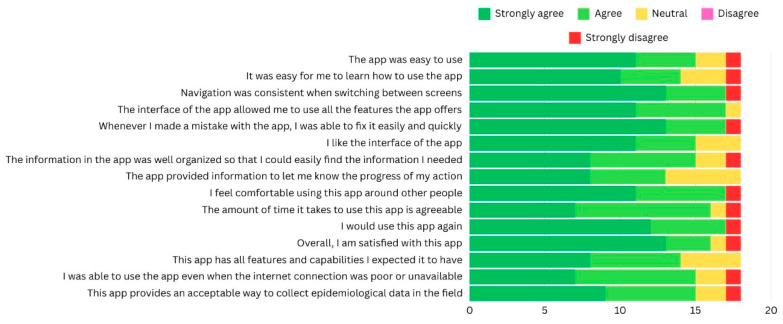
Clinicians’ responses to modified mHealth app usability questionnaire [22].

The 18 clinicians who used the SECAT application completed the questionnaire, providing a 100% response rate. The aggregated weighted score revealed an overall 82% satisfaction toward using the SECAT application. Figure 5 illustrate the clinicians’ responses satisfaction questionnaire. The vast majority of clinicians (78%, n = 14) strongly endorsed navigation throughout the application and ease of use. They specifically highlighted its user-friendly characteristics, including intuitive navigation, straightforward learning curve, seamless interface design, error recovery capabilities, and overall ease of use. Based on these positive experiences, clinicians expressed strong intention to continue utilizing the application in their practice.

In contrast, the primary concerns raised were related to color contrast preferences for distinguishing between different coding systems and dental characteristics. Additionally, participants expressed a preference for the inclusion of individual-level calculations of decayed, missing, and filled teeth (DMFT index) per patient.

In terms of suggestions for improvement, participants expressed several modifications to the diagnosis and charting interface. Specifically, they recommended implementing a dual-column layout for diagnostic options, rather than the current arrangement, to improve visual clarity and navigation. Furthermore, to optimize the reporting process, participants suggested incorporating an automated default setting whereby unexamined teeth would be automatically classified as sound, thus streamlining the documentation workflow and reducing redundant data entry requirements.

#### 3.3.3. Heuristic Study Results

The panel of five experts identified a significant technical limitation in the application’s inability to distinguish between primary, permanent, and mixed dentition. To address this issue, they proposed implementing an auto-identification feature based on tooth selection.

Despite this limitation, the experts highlighted several positive aspects of the application. They noted its utility in remote areas, emphasizing its offline functionality, which makes it especially valuable for community dentistry and field-based dental screening. The application’s user-friendly interface and clear design were also commended. Furthermore, the experts recognized its potential to streamline the process of recording findings, transitioning from paper-based methods to a more efficient data management system. They also noted that the application’s data retrieval capabilities could enhance research efforts and decision-making processes while potentially reducing reporting errors.

In terms of technical improvements, the experts offered three main suggestions. Firstly, they recommended the coloring system to be more prominent through coloring the whole tooth instead of outlining it only. Secondly, they suggested enhancing the application’s functionality by including the presentation of key clinical information, such as calculated dmft (decayed, missing, and filled teeth) indices for both individuals and assigned groups. Examples of clinicians’ and experts’ feedback of post-usability test is provided in Appendix E.

### 3.4. System Enhancements Based on User-Centered Evaluation 

Based on comprehensive usability and acceptability studies involving both clinicians and dental experts, several modifications were implemented to enhance the system’s functionality. The modifications addressed three primary areas of improvement:

1. Automated Tooth Recognition System

The integration of an automated tooth identification feature was implemented, enabling precise recognition based on individual tooth selection. This enhancement significantly improved the accuracy of dental charting and reduced manual input requirements.

2. Enhanced Visualization Protocol

**Figure 6:**
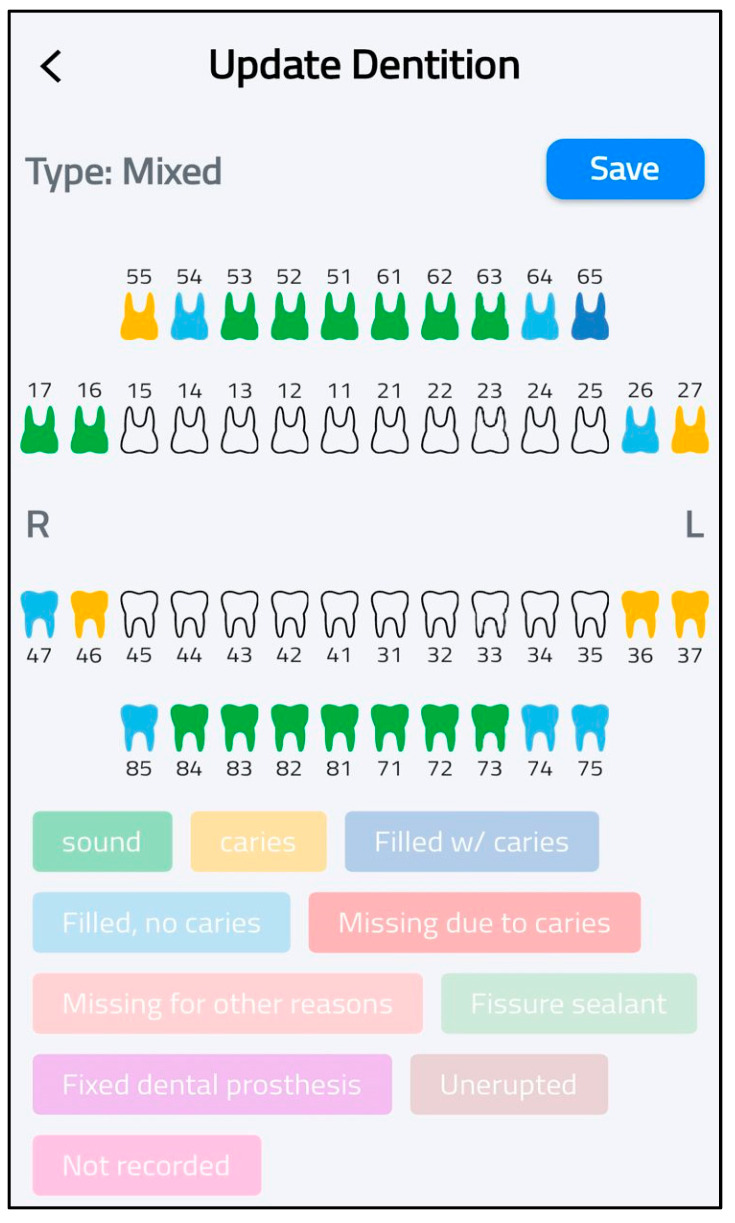
Enhanced visualization protocol.

The visualization system was revised from an outline-based approach to a complete tooth-surface coloring methodology. This modification provided improved visibility and clarity in dental status representation, facilitating more accurate clinical assessment and documentation (Figure 6).

3. Clinical Metrics Integration

**Figure 7:**
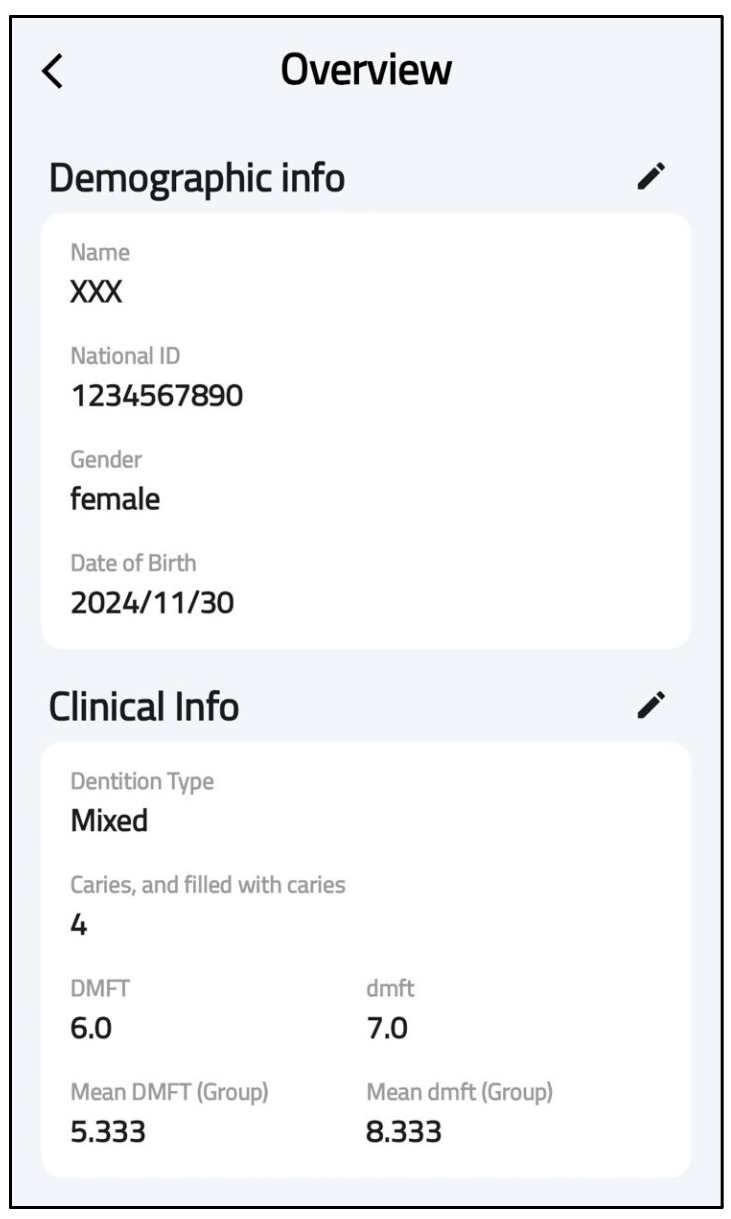
Clinical metrics integration.

A significant enhancement involved the incorporation of essential clinical parameters, specifically the dmft (decayed, missing, and filled teeth) indices. The system now generates both individual and group-level dmft scores, enabling comprehensive analysis of dental health status across different patient cohorts (Figure 7).

These modifications were implemented in response to structured feedback obtained through systematic evaluation by dental clinicians and experts in the field.

## 4. Discussion

### 4.1. Principal Findings

The current feasibility study aimed to develop and evaluate the impact, usability, and acceptability of a SECAT mobile application for collecting dental examination data in remote locations from the perspectives of dental health providers (dentists and dental hygienists). Dental health professionals lacked experience with dental assessment applications and were unaware of available dental charting tools. However, they appreciated the potential benefits that applications may have in remote clinical settings. The SECAT mobile application was developed, considering the preferences of dental health providers, the need to standardize dental data collection in the field/practice, and the need to enable real-time data transmission.

It was suggested to implement a dual-column layout for diagnostic options to improve visual clarity and navigation, in addition to incorporating an automated default setting whereby unexamined teeth were classified as sound. These suggestions were considered and incorporated to enhance the usability of the SECAT. In terms of technical enhancement, the coloring of whole tooth and inclusion of key clinical information to calculate dmft was implemented.

Besides the limitations raised by the experts, several positive aspects were also identified, particularly the utility of SECAT application in remote areas, emphasizing its functionality in offline areas.

### 4.2. Comparison with Prior Work

In the literature, there are several applications developed for promoting oral health and improving oral health knowledge and behaviors [23,24,25,26,27,28]. On the other hand, few studies have developed and evaluated the usability of applications related to oral health survey [29,30,31].

A study conducted in Brazil between 2018 and 2019 developed and presented the field performance of a software (NutriOdonto https://github.com/bcunhasa/nutriodonto (accessed on 11 December 2024)) for conducting an epidemiological caries survey. NutriOdonto is a software developed for the collection, analysis, management and reproducibility of oral health epidemiological research among 9th grade elementary school students. The software presented, among other features, the exemption of the data transfer step recorded for a computer, thus contributing to the reduction in information distortion, generating more reliable and faster data. Furthermore, NutriOdonto enabled the application of two structured electronic questionnaires (individual, behavioral and socioeconomic characteristics of students, and sociodemographic characteristics of schools) [29]. 

Another study conducted in Thailand aimed to develop Oral Health Survey Mobile Application (OHSMA), to analyze the oral status of grades 1–6 Thai school children using OHSMA, and to evaluate user satisfaction of using OHSMA. This android application was developed to facilitate the collection and transfer of oral health status data. The main study findings indicated high satisfaction scores for three OHSMA features: font, color, and proper size. The results showed that paper forms were easier for inputting and recording the data compared with the OHSMA. However, the OHSMA was significantly easier for searching data and reporting data compared to paper forms. Unfortunately, OHSMA is an android application, so iOS users could not access it through their mobile devices and could access it only through a web-based platform, which requires internet connection, thus limiting its availability [30].

Recently, Tooth Memo was developed, which is an improved version of OHSMA and is compatible with both android and iOS devices to digitize oral health survey data and personal oral health information. Tooth Memo can be used when internet connection is unavailable, which makes it useful to use in field projects conducted in rural areas. This application offers several features including calculating the mean DMFT index, providing notification if the examination is incomplete or complete, survey forms for dental fluorosis and prosthesis status. The study found that transferring data from paper to a computer took an average of 55 s per case. Among 103 cases, the manual method required 182 min more than the iOS or android methods to clean missing data, and transfer and analyze the tooth status data. The users, who were fifth-year dental students and laypeople, reported high satisfaction with using the Tooth Memo application [31].

In Saudi Arabia, dental caries prevalence reached up to 100% in primary teeth and 99% in permanent teeth between 1999 and 2019 [5]. Access to oral healthcare services is limited in remote and underserved areas, which exaggerates the negative impact of dental caries. Moreover, conducting oral health surveys in remote locations poses significant logistical challenges related to equipment transportation, data recording and transmission. Additionally, informing public health interventions and resource allocation requires accurate and timely data collection on dental caries. Therefore, with the advancement and growing integration of mobile technologies in healthcare systems, there is an emerging need for mobile applications to enhance epidemiologic surveillance in dentistry. While several applications developed for promoting oral health and improve oral health knowledge and behaviors, few solutions have been specifically designed for oral health providers to conduct caries assessments in resource-limited settings. This study aimed to address this gap by developing and evaluating the SECAT, a mobile application designed to standardize caries data collection, improve efficiency, and enable real-time data transmission from field sites to centralized databases in remote locations in Saudi Arabia.

### 4.3. Limitations

We identified several key limitations in this study. First, the utilization of convenience sampling for dental healthcare providers may impact the external validity and generalizability of our findings. As noted in qualitative design research [32], while sample sizes are typically small compared to traditional academic research, speaking with 5 to 10 representative participants is often sufficient to uncover common challenges, understand underlying causes, and inform decisions. Secondly, SECAT’s current developmental status and limited accessibility restricted our ability to conduct broader-scale analysis with a more diverse participant pool. Expanding the evaluation to a broader expert pool would strengthen the robustness of the findings and provide a more comprehensive understanding of SECAT’s usability. Thirdly, the learning effect must be considered when interpreting the results as the participants were first-time SECAT users. This consideration is particularly relevant in digital health intervention (DHI) evaluation, where user familiarity can significantly impact outcomes. Finally, potential confounding variables may have influenced the study findings, highlighting the need for more controlled investigations in future research phases. As is common in early-stage DHI research, these limitations could be addressed through iterative testing and expanded user engagement in subsequent development phases.

### 4.4. Future Directions

Future research should include a larger sample size to improve the validity and reliability of the current results. Therefore, more longitudinal studies comprising nation-wide and global population are necessary to evaluate the long-term impact of the application and the study outcomes. Furthermore, compiling of SECAT application in terms of data exchange as per the local and international standard needs consideration.

### 4.5. Practical Implications

The SECAT mobile application could be valuable aid for caries assessment in remote places and also in school and community based dental health programs. The application will promote technologically advanced, user-friendly, paperless and more importantly cost-effective process of recording dental caries in accordance with WHO recommended guidelines.

## 5. Conclusions

The use of the SECAT application for collecting dental caries data showed a high satisfaction and acceptability among a group of dental health providers. The user-friendly characteristics, including intuitive navigation, straightforward learning curve, seamless interface design, error recovery capabilities, and overall ease of use of the SECAT application were specifically highlighted. The preliminary evaluation identified several limitations with possible solutions and identified positive aspects in terms of SECAT application’s utility in areas with limited internet connection.

## Figures and Tables

**Table 1:** Participants characteristics at the ‘requirements gathering’ step (n = 12).

Characteristics	N (%)
Gender	
Male	4 (33)
Female	8 (67)
Age group	
25–29	4 (33)
30–39	5 (43)
40–49	2 (16)
50–59	1 (8)
Profession	
Dentist	6 (50)
Dental Hygienist	6 (50)
Work Experience (years)	
2–5	2 (16)
6–10	7 (59)
11–15	3 (25)
Smartphone owner	
Yes	12 (100)
No	0 (-)
Smartphone brand	
iPhone	10 (84)
Samsung	2 (16)
Others	0 (-)

**Table 2:** Participants characteristics at the evaluation step.

	Clinicians	Experts
Characteristic	N (%)	N (%)
**Gender**		
Male	8 (44)	2 (40)
Female	10 (56)	3 (60)
**Age group**		
25–29	4 (22)	0 (-)
30–39	6 (33)	1 (20)
40–49	5 (28)	4 (80)
50–59	3 (17)	0 (-)
**Profession**		
Dentist	9 (50)	3 (60)
Dental Hygienist	9 (50)	2 (40)

**Figure A1:**
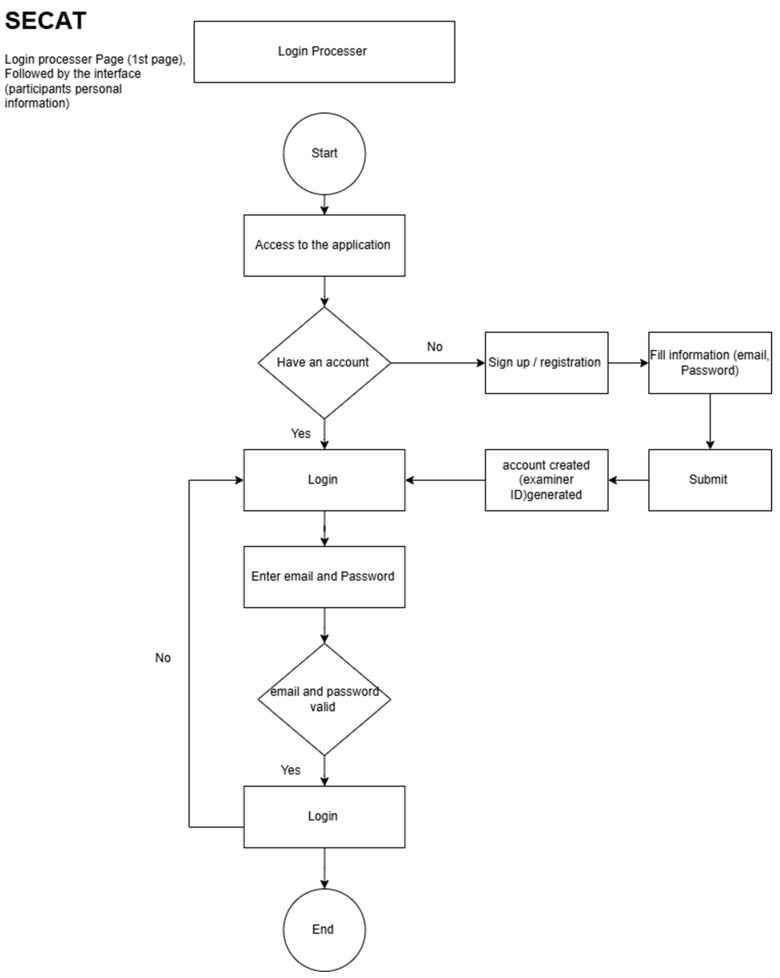
Page 1 SECAT flowchart.

**Figure A2:**
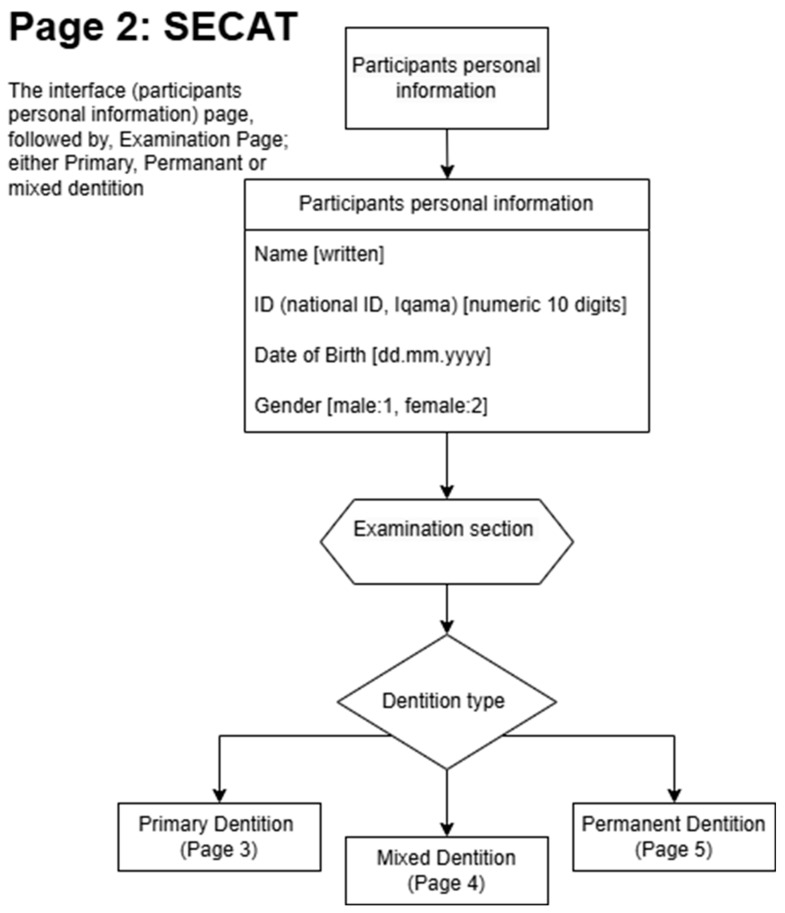
Page 2 SECAT flowchart.

**Figure A3:**
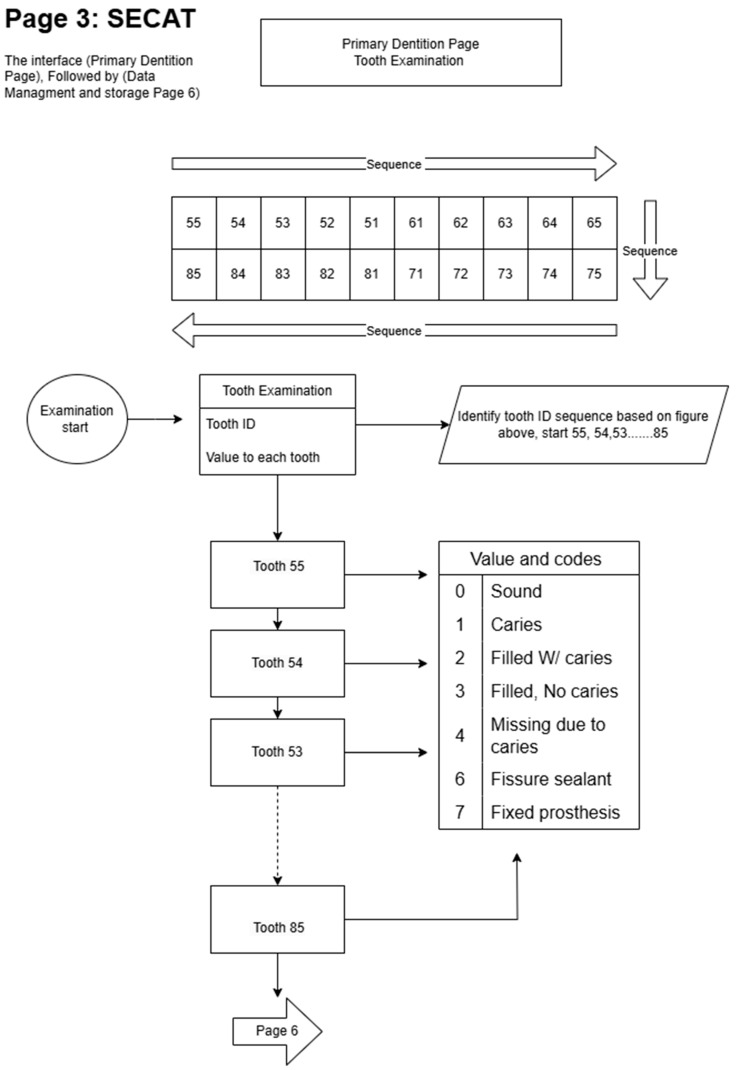
Page 3 SECAT flowchart.

**Figure A4:**
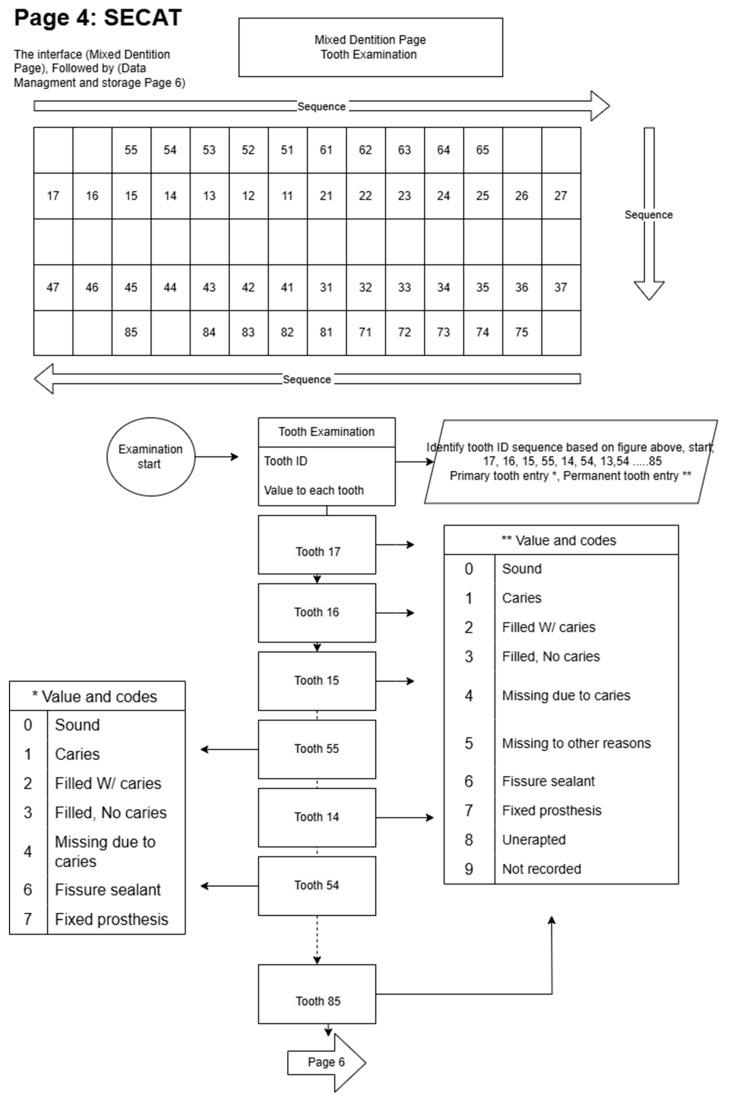
Page 4 SECAT flowchart. * Primary teeth value and codes, ** Permanent teeth value and codes.

**Figure A5:**
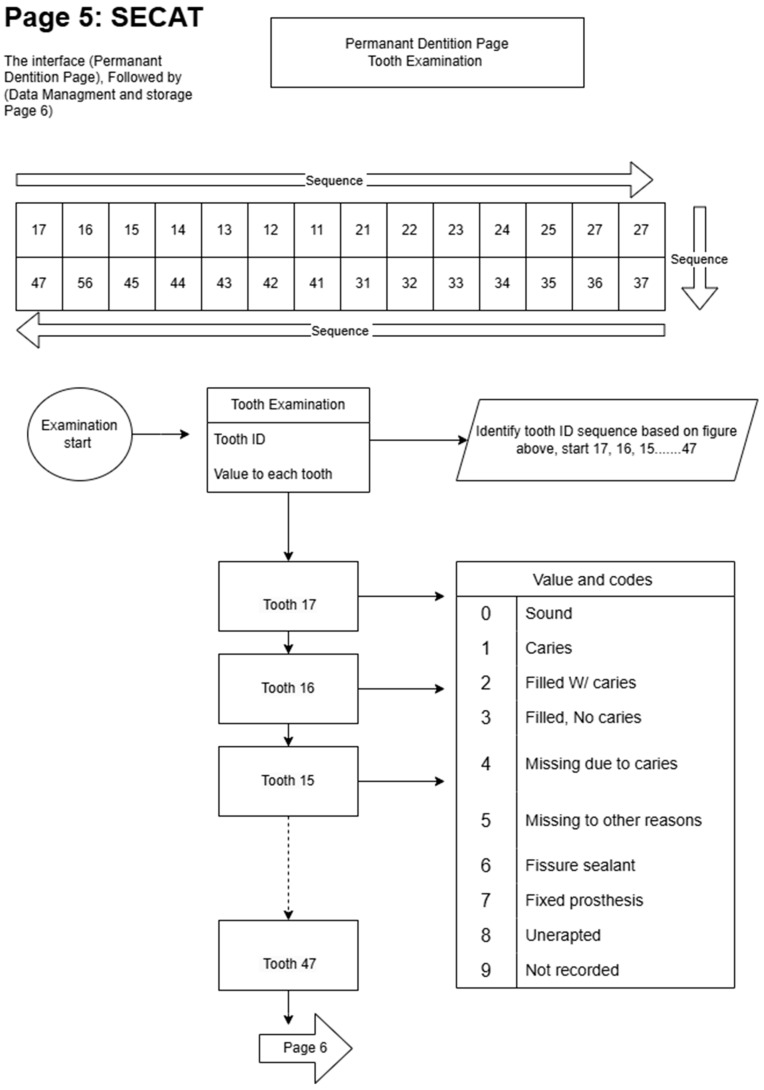
Page 5 SECAT flowchart.

**Figure A6:**
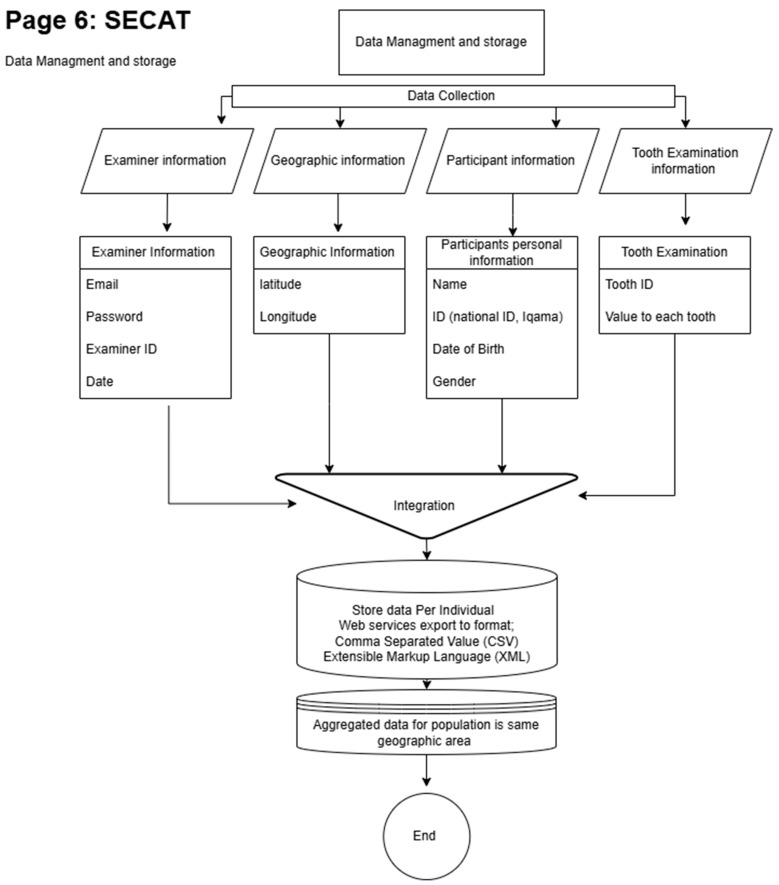
Page 6 SECAT flowchart.

## Data Availability

The datasets used and/or analyzed during the current study are available from the corresponding author on reasonable request.

## Abbreviations

mHealth	Mobile health applications
SECAT	Saudi Electronic Caries Assessment Tool
WHO	World Health Organization
IRB	Institutional Review Board
KKUH	King Khalid University hospitals
CAMS	College of Applied Medical Sciences
NVivo	Non-Versioned Information, Versatile Outcomes, an application used to analyze qualitative and mixed methods research
API	Application Programming Interface
HTTPS	Hypertext Transfer Protocol Secure
OHSMA	Oral Health Survey Mobile Application
iOS	iPhone Operating System

## Appendix B. Satisfaction Questionnaire

Evaluating Usability of SECAT Using Modified Version of mHealth App Usability Questionnaire (MAUQ) [22]

**Questions**	**Strongly** **Disagree**	**Disagree**	**Neutral**	**Agree**	**Strongly** **Agree**
1. The app was easy to use	□	□	□	□	□
2. It was easy for me to learn how to use the app	□	□	□	□	□
3. Navigation was consistent when switching between screens	□	□	□	□	□
4. The interface of the app allowed me to use all the features the app offers	□	□	□	□	□
5. Whenever I made a mistake with the app, I was able to fix it easily and quickly	□	□	□	□	□
6. I like the interface of the app	□	□	□	□	□
7. The information in the app was well organized so that I could easily find the information I needed	□	□	□	□	□
8. The app provided information to let me know the progress of my action	□	□	□	□	□
9. I feel comfortable using this app around other people	□	□	□	□	□
10. The amount of time it takes to use this app is agreeable	□	□	□	□	□
11. I would use this app again	□	□	□	□	□
12. Overall, I am satisfied with this app	□	□	□	□	□
13. This app has all features and capabilities I expected it to have	□	□	□	□	□
14. I was able to use the app even when the internet connection was poor or unavailable	□	□	□	□	□
15. This app provides an acceptable way to collect epidemiological data in the field	□	□	□	□	□

## Appendix C. Two Simulation-Based Case Scenarios

*1st Scenario*

Open the application

Register your info

Add a group (School1)

Add participant to the group

Name: Haya M A

Date of Birth: 16 August 2022

Gender: Female

ID: 1012345678

Please add tooth description as following:

#17	#16	#55	#54	#53	#52	#51	#61	#62	#63	#64	#65	#26	#27
Unerupted	caries	filled with caries	Sound	Sound	Sound	Sound	Sound	Sound	Sound	Sound	caries	caries	Unerupted
Unerupted	FS	Filled no caries	Filled no caries	Sound	Sound	Sound	Sound	Sound	Sound	Filled no caries	Filled no caries	FS	Unerupted
#47	#46	#85	#84	#83	#82	#81	#71	#72	#73	#74	#75	#36	#37

Save the data

Sign out

*2nd Scenario*

Log in using your info

Choose School 1

Add new Participant

Name: Arwa A T

Date of Birth: 13 May 1990

Gender: Female

ID: 1087654321

Please add tooth description as following:

#17	#16	#15	#14	#13	#12	#11	#21	#22	#23	#24	#25	#26	#27
FS	Filled no caries	Filled no caries	Sound	Sound	Sound	Sound	Sound	Sound	Sound	Sound	Filled no caries	Filled no caries	FS
FS	FS	Filled no caries	Filled no caries	Sound	Sound	Sound	Sound	Sound	Sound	Filled no caries	Filled no caries	FS	Filled no caries
#47	#46	#45	#44	#43	#42	#41	#31	#32	#33	#34	#35	#36	#37

Save the participant

Download the data through going back to groups → press the icon on top left side select (Download my data)

Sign out
